# Quality control of CT systems by automated monitoring of key performance indicators: a two‐year study

**DOI:** 10.1120/jacmp.v16i4.5469

**Published:** 2015-07-08

**Authors:** Patrik Nowik, Robert Bujila, Gavin Poludniowski, Annette Fransson

**Affiliations:** ^1^ Department of Medical Physics Karolinska University Hospital Stockholm Sweden

**Keywords:** computed tomography, quality assurance, quality control, key performance indicators, image analysis

## Abstract

The purpose of this study was to develop a method of performing routine periodical quality controls (QC) of CT systems by automatically analyzing key performance indicators (KPIs), obtainable from images of manufacturers' quality assurance (QA) phantoms. A KPI pertains to a measurable or determinable QC parameter that is influenced by other underlying fundamental QC parameters. The established KPIs are based on relationships between existing QC parameters used in the annual testing program of CT scanners at the Karolinska University Hospital in Stockholm, Sweden. The KPIs include positioning, image noise, uniformity, homogeneity, the CT number of water, and the CT number of air. An application (MonitorCT) was developed to automatically evaluate phantom images in terms of the established KPIs. The developed methodology has been used for two years in clinical routine, where CT technologists perform daily scans of the manufacturer's QA phantom and automatically send the images to MonitorCT for KPI evaluation. In the cases where results were out of tolerance, actions could be initiated in less than 10 min. 900 QC scans from two CT scanners have been collected and analyzed over the two‐year period that MonitorCT has been active. Two types of errors have been registered in this period: a ring artifact was discovered with the image noise test, and a calibration error was detected multiple times with the CT number test. In both cases, results were outside the tolerances defined for MonitorCT, as well as by the vendor. Automated monitoring of KPIs is a powerful tool that can be used to supplement established QC methodologies. Medical physicists and other professionals concerned with the performance of a CT system will, using such methods, have access to comprehensive data on the current and historical (trend) status of the system such that swift actions can be taken in order to ensure the quality of the CT examinations, patient safety, and minimal disruption of service

PACS numbers: 87.57.C‐, 87.57.N‐, 87.57.Q‐

## I. INTRODUCTION

The number of CT examinations performed worldwide and the high doses associated with those CT examinations makes up‐to‐date information on the performance of CT systems a prerequisite to ensure patient safety and the quality of examinations. The total time required and the limited access to the CT scanners often restrict the possibilities for the medical physicist to perform quality control (QC) at more than the recommended annual frequency.^(1)^ A CT scanner is likely to undergo preventive maintenance, hardware and/or software upgrades, and repairs between annual QC. In addition, degrading and/or malfunctioning of the hardware can occur and might go undetected until the next maintenance or annual routine QC. Major performance abnormalities are likely to be detected by technologists during routine work, but more subtle issues may go unnoticed.

One of the main responsibilities of a medical physicist is to establish, implement, and supervise a quality assurance (QA) program.^(2)^ Today, most QC procedures, apart from being performed only annually, contain subjective elements (e.g., the manual positioning of ROIs). This reduces the reproducibility of consecutive QC tests. Larger uncertainties and low frequency of testing reduce statistical power in trend analysis, rendering it less valuable. A modern routine QC should ideally have fully automated evaluation routines and store the results in a database.^(3)^ The results will then be at their most reliable, reproducible, and useful for long‐term trend analysis.

More frequent QC of CT systems in order to ensure scanner performance and acceptable image quality has been suggested by the American College of Radiology (ACR). ACR also state that automated QC procedures may be used if they are reviewed correctly. In its 2012 CT QC report, ACR recommends daily QC, including monitoring the CT number of water, the image noise, and image artifacts.^(1)^ IEC, instead, recommends that the noise, CT number of water, and image uniformity should be evaluated monthly.^(4)^ Many sites around the world already have daily QC program where CT technologists perform a limited number of tests. When automatic evaluation procedures are lacking, however, recommendations such as those of the ACR can be difficult to implement in clinical practice. The aim of this work was to develop a methodology and software tools to enable a comprehensive raft of QC tests to be carried out rapidly on a daily basis with minimal operator intervention. More than a simple logging of QC results, the system provides automated alerts to physics staff and the facility for trend analysis. The advantages of such an automated QA system is that performance issues may be identified earlier without unduly increasing demands on CT technologists, and it also allows physicists to implement more sophisticated analysis of images. It also provides insight into the behavior of CT scanners on a day‐to‐day basis and assists in identifying trends in slow performance degradation. While the burden for a CT technologist between a nonautomated and an automated QA system is not necessarily large, the results from an automated QA system are more valuable, since operator variability is removed. Further, in addition to a basic set of tests, physicists have the option of including more sophisticated tests without an increase in burden to the technologist.

We note that some manufacturers have their own automated QC routines incorporated in their CT systems. The results from the QC performed with such routines are not typically easily accessed and it is, therefore, hard to perform trend analysis on the data. In this study we sought to develop analyses that are vendor‐independent. An important part of this work was to identify key performance indicators (KPIs) in CT. A KPI pertains to a measurable or determinable parameter that is influenced by other underlying more fundamental parameters affecting scanner performance. While the established QA program with annual QC consists of an extensive set of independent parameter tests, a QC of KPIs alleviates the intricacies involved with performing such “full‐blown” QC. The KPI method offers a simplified routine and makes periodic QC accessible while maintaining insight into most parameters that are tested annually. Furthermore, when a KPI deviates, the deviation can be systematically located by following the KPIs dependencies. Relevant (independent) tests can then be performed to isolate the real origin of the problem. By introducing such a simplified yet comprehensive test, the performance of CTs can be sampled at tighter intervals and complement the annual QC test.

Once the KPIs were identified in this work, the methodology was implemented as a daily QC program on two of the CT scanners at the Karolinska University Hospital in Stockholm, Sweden, using the manufacturer's QA phantom. Results from this daily QC program during a two‐year period are presented.

## II. MATERIALS AND METHODS

### A. Key performance indicators (KPIs)

A KPI pertains to a measurable or determinable parameter that is influenced by other underlying parameters. A stable KPI implies that the underlying parameters are stable as well. To perform daily QC procedures that cover as many parameters as possible from routine annual QC, KPIs were identified and verified by studying and mapping out the relationship between established QC parameters used in annual testing.

The KPIs include positioning, image noise, uniformity, homogeneity, CT numbers of water, and CT number of air. The KPI positioning needs a dedicated positioning part in a manufacturer's phantom, while the others are determined from images of the water part. The available QC procedures of various parameters in a QA program,^5^, ^6^, ^7^, ^8^, ^9^ and the KPIs main dependencies on QC parameters are illustrated in Table 1

**Table 1:** CT parameters that generally are available with a CT QA program, how often they should be tested, and on which KPIs they mainly depend

*Parameters Tested During QA* (A=acceptance,C=constancy)		*QA Parameter Dependencies on KPIs*	
*Direct Tests*		*Positioning*	*CT Numbers*
LASER accuracy (A, C)	Uniformity (A, C)	Table top indexing	CT number of water
Table top indexing (A)	Inter‐slice noise (A, C)	Table top orientation	CT number of air
Table top orientation (A)	Geometric accuracy (A)	Scan plane localization	Calibration
Scan plane localization (A, C)	Spatial resolution (A, C)		
CTDI_vol_ (A)	Scattered radiation (A)	*Image noise*	*Uniformity*
CTDI_air_ (A, C)	System documentation (A, C)	X‐ray tube voltage	Shaped filter
		CTDI_vol_	Reconstruction
Dose‐noise response (A)	Radiation protection	CTDI_air_	Detector
Dose‐noise linearity (A)	documentation (A, C)	Spatial resolution	
X‐ray tube voltage (A, C)	Radiation shielding (A, C)	HVL	*Homogeneity*
HVL (A, C)	Radiation notification (A, C)	Low contrast resolution	Artifact evaluation
Z‐dose profile (A)	Staff interview (C)	Collimation	
Geometric efficiency (A)	Artifact evaluation (A,C)	Slice thickness	
CT number of air (A, C)		Geometric efficiency	
CT number of water (A, C)	*Indirect tests*	Z‐dose profile	
CT numbers of various materials (A, C)	Shaped filter	Dose‐noise response	
CT number linearity (A, C)	Calibration	Detector response	
Slice thickness (A, C)	Dose display (A,C)	Inter‐slice noise	
Image noise (A, C)			

#### A.1 Positioning

The primary goal with the positioning test is to assess the CT scanner's ability to correctly position the object being imaged. From a clinical view point this is important as it has been shown that patient miscentering may lead to both higher surface doses and noisier images.^(10)^ Positioning depends on the precision of the gantry lasers and the functionality of the tabletop of the CT scanner. Positioning that is out of tolerance may have the consequence that other KPI measurements cannot be fully trusted since they are calculated with the phantom miscentered.

The KPIs for positioning are the values of the X, Y, and Z offsets of the phantom. The manufacturer's phantom usually includes a positioning part where these parameters can be calculated.

The tolerances for the positioning lasers for the X, Y, and Z offset were chosen as ± 2 mm. This was in accordance with the tolerances defined by the manufacturer and is also what the Nordic Association of Clinical Physics (NACP) and ACR recommend.^1^, ^11^

#### A.2 Image noise

The image noise KPI is influenced by all parameters that can affect the pixel values in an image, parameters related to the X‐ray tube output and the reconstruction. The Brooks formula^(12)^ (reformulated by Nagel^(13)^) shows that image noise (σ) varies with the attenuation of the object, radiation dose, pixel size, beam collimation, and slice thickness. It can be expressed as

(1) $$\sigma^{2}\propto \frac{e^{-\mu \cdot d}}{D\cdot b^{2}\cdot w\cdot T}$$

where *μ* is the linear attenuation coefficient, *d* is the thickness of the object with attenuation $\mu ,e^{-\mu \cdot d}$ is the attenuation of the object, *D* is the CT dose index ${\text{CTDI}}_{\text{VOL}}$, $b^{2}$ is the sampling distance at the rotation center (pixel size), *w* is the beam collimation, and *T* is the slice thickness. The image noise dependence on dose can be expanded by taking into account that the dose varies linearly with tube current and with exposure time. In addition, image noise dependence on tube peak voltage can be introduced since the radiation dose varies as

(2) $$D\propto kVp^{n}$$

where *kVp* is the peak voltage and *n* is a kilovoltage dependency factor that has been estimated to have a value of approximately 2.6.^(14)^ The value *n* depends on the radiation quality of the beam, tube output, and detector response. The Brooks formula can be rewritten by inserting the tube voltage (kVp), tube current (mA), and exposure time (s) dependences on dose, yielding

(3) $$\sigma^{2}\propto \frac{e^{-\mu \cdot d}}{mA\cdot s\cdot kVp^{n}\cdot b^{2}\cdot w\cdot T}$$

A base value of the KPI image noise is defined at the acceptance testing of the CT scanner and is used as a comparison in future tests. The tolerance for the variation of the image noise that is applied at the annual quality controls of the CT scanners at the Karolinska University Hospital is set to 10% of this base value. This tolerance is the same as recommended by the NACP and IEC.^4^, ^11^ The automated image analysis used in this work allows, however, for a constant and reproducible ROI diameter of 40% of the phantom^(6)^ diameter at every test. Initial tests showed that the automatization of ROI definition allowed the use of a lower tolerance. A reduction to 5% of the base value for the image noise was tested and implemented in this work. As a lowered tolerance for the variation in image noise translates into less variation possibilities for the underlying parameters, the test will pick up bad machine performance earlier than what otherwise would be possible.

The effect on this KPI when an underlying parameter is out of tolerance can be evaluated directly from the Brooks formula

(4) $$\frac{\sigma_{new}}{\sigma_{old}}=\sqrt{\frac{D_{old}}{D_{new}}}$$

The relation shows how the image noise will change depending on variations in dose. In order to use this expression to monitor changes of underlying parameters by measuring the noise, it is of importance to use the same scan parameters at every QC procedure.

According to the IEC^(4)^ if both CT numbers and image noise are within specified tolerances, the specified low‐contrast resolution is also expected to be within tolerance and, for that reason, specific tests for low‐contrast resolution are not performed with this method. The magnitude of image noise is sensitive to small variations in the spatial frequency distribution of the MTF in the reconstruction of images.^15^, ^16^ Therefore, if a specific reconstruction algorithm produced images with slight changes in its MTF, it could possibly be discovered with the KPI image noise.

#### A.3 CT number of water and air

According to the definition of the Hounsfield unit (HU), air (approximately vacuum) has a CT number of −1000 HU and water has a CT number of 0 HU. These two values provide two calibration points that affect the CT number of all other materials. These KPIs are mainly influenced by the scanner's reconstruction algorithm and its calibration. Although they are also influenced by the X‐ray spectra and the object attenuation, the impact of those aspects are covered by other KPIs. Given that the manufacturer's phantom is used both for the CT scanner calibration and the QC tests, changes in the air and water CT number KPIs are expected to directly reflect any change in CT scanner calibration

The automated measurements of the CT number is performed with a ROI diameter that is 10% of the phantom diameter.^(6)^ The CT number tolerance used during annual quality controls at the hospital is ± 4 HU for water and ± 10 HU for air. These are the same values as those recommended by the NACP and IEC.^4^, ^11^ As in the case with image noise discussed in the previous section, the automated definition of the ROI in this work resulted in a very stable estimate of the CT number of water and a reduction of the tolerance was possible. The tolerance for CT numbers of water was empirically chosen to be ± 2 HU. The same reduction could not be applied for air since the ROI for air were put outside the phantom. This is contrary to the recommendation of the IEC, and resulted in increased variability with respect to water. Hence, the tolerance for CT numbers of air was kept at ± 10 HU. The chosen tolerances can be compared to the tolerances suggested by the vendor, which are ± 3 HU for water and ± 10 HU for air.

#### A.4 Uniformity

The uniformity KPI is a test that evaluates the CT scanner performance in reconstructing a uniform image. The uniformity of the image depends on the shaped beam filter, X‐ray tube output, and on the centering of the object in the beam. The test focuses on the capability of the CT scanner to produce an image free from beam hardening and artifacts.

The placement of ROIs in this test includes one ROI that is positioned in the center of the phantom and four ROIs that are placed in the periphery of the phantom and located at 3, 6, 9, and 12 o'clock. The peripheral ROIs are placed 1 cm from the edge.^(6)^ In order to exclude analyzing pixels in which the CT number might have an appreciable influence from the PMMA housing of the phantom, the center of the peripheral ROIs is located at a distance corresponding to 15% of the phantom diameter away from the edge of the phantom. The ROIs have a diameter that corresponds to 10% of the phantom diameter.^(11)^ Following the recommendations of the IEC, image uniformity (an indicator of the existence of beam hardening effects) was evaluated as the maximum difference between the mean value of the center ROI and any of the four peripheral ROIs.^(6)^

The tolerance for the variation of image uniformity used in the annual quality controls at the Hospital is 4 HU according to NACP recommendations.^(11)^ In this work, a tolerance of 3 HU was used (as suggested by the manufacturer).

#### A.5 Homogeneity

**Figure 1:**
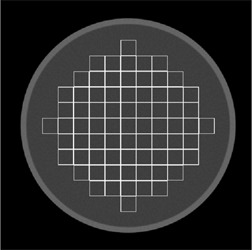
Placement of ROIs of the KPI homogeneity test.

The KPI homogeneity was introduced in order to increase the likelihood of identifying artifacts associated with degradations of CT scanner performance (e.g., ring artifacts and local nonuniformities caused by air bubbles in the cooling oil). Contiguous rectangular 32×32 pixel ROIs are used to cover a circular area with a diameter of 85% of the diameter of the homogenous water phantom section of the manufacturer's QA phantom. An illustration of the ROI placement is shown in Fig. 1. The size of the area covering the phantom was chosen in order to exclude analyzing pixels in which the CT number might have an appreciable influence from the PMMA housing. This does mean, however, that artifacts may go undetected if they arise in the area outside the covered area. The size of the individual ROIs was empirically determined with the aim to provide a good tradeoff between being too large (artifacts can go undetected) and being too small (false positives can be found due to the statistical nature of CT numbers). The KPI homogeneity is defined as the maximum difference in mean CT number between any of the ROIs.

This performance test for a CT scanner is not included in any standard QA procedure and has, to our knowledge, not been suggested before. As the homogeneity test is an extension of the concept of the uniformity test, it has the potential to replace the latter. Currently we have chosen to keep the uniformity KPI as it is recommended by the IEC to perform a uniformity test at least monthly.^(4)^ The homogeneity tolerance level is still undergoing refinement. The approach for choosing the tolerance was to select a first tolerance level that from initial tests seem reasonable and then, as more data are collected, systematically lower the tolerance. The opposite approach, to start with a low tolerance and systematically increase the tolerance could have been chosen, but would have generated more false positives. The first tolerance level that was used was 6 HU (larger than the tolerance of uniformity) and following the first year of tests, the tolerance was lowered to 5 HU.

### B. Methodology of routine QC by monitoring of KPI

A method of performing routine QC procedures of CT scanners in terms of the identified KPIs was developed. The method relies on imaging the manufacturer's QA phantom. Two imaging protocols were defined. The first protocol (Level 0) was used as a standard test of the KPIs. The second protocol (Level 1) was set up to systematically search for the underlying fundamental QC parameter that caused the KPI to deviate out of tolerance. The Level 0 test includes a single slice over the reference markings of the manufacturer's QA phantom, together with one scan (having multiple slices) over the homogenous water section of the phantom. If the Level 0 passes, the CT scanner fulfills its performance requirements for clinical use.

If a Level 0 test fails, the more comprehensive Level 1 test is required. In the same way as for the Level 0 test, the Level 1 test is performed by scanning the manufacturer's QA phantom. This test includes imaging of the same single slice as in Level 0 (used to evaluate the KPI for positioning), together with a series of multiple scans over the homogenous water section of the manufacturer's QA phantom. For each scan in the Level 1 test, imaging parameters that the different KPIs depend on are varied systematically in order to locate which of the fundamental QC parameters caused the KPI(s) to deviate from tolerance.

**Figure 2:**
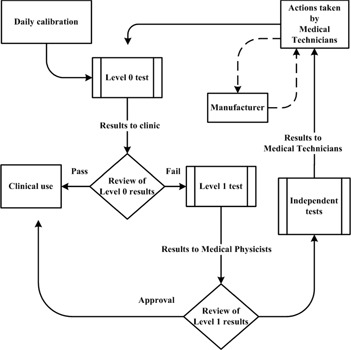
Schematic illustration of the proposed routine QC methodology.

It is the medical physicist's task to analyze why a QC has failed, with the combined data from the Level 0 and Level 1 tests. Following this analysis, the medical physicist has a good starting point for performing any additional and more focused tests (with, for example, dedicated phantoms or other instruments). Once the fundamental QC parameter causing the problems has been verified, efforts to service the CT scanner promptly can be initiated. The proposed methodology of routine QC by monitoring KPIs is illustrated in Fig. 2.

In order to make an efficient implementation of the procedure, a software application, MonitorCT, was developed in‐house in order to receive the set of KPI scans and to automatically analyze the images. The results from the analyses are stored in a database and tested against the defined tolerance levels. The imaging protocols have been configured to transfer the acquired images automatically to the software. As the procedure now is almost entirely automated, the time needed for the tests to be performed and evaluated has greatly decreased.

For the CT technologist, the testing procedure consists of: (i) mounting the manufacturer's QA phantom, (ii) positioning the phantom according to the CT positioning laser and the phantom markings, and (iii) selecting the predefined protocol for the desired test (Level 0 or Level 1). The time required by the CT technologist to perform a test, from mounting of the phantom to a report of the results, is approximately 2 min for the Level 0 test and approximately 3 min for the Level 1 test.

### C. Pilot study

The method for performing routine QC proposed in the Material & Methods section B using the set of KPIs derived in section A was tested on two CT scanners at the Hospital over a period of two years. The CT scanners included a GE LightSpeed VCT (GE Healthcare, Waukesha, WI) and a GE Discovery CT750HD (GE Healthcare, Waukesha, WI). Both scanners are installed at the Department of Neuroradiology at the Karolinska University Hospital. The GE LightSpeed VCT and the GE Discovery CT750HD are subsequently referred to as CT1 and CT2, respectively, in this work. Due to the unprecedented nature of the methodology, we chose to perform the tests on a daily basis where a Level 0 test was performed Monday to Thursday and a Level 1 test was performed every Friday (and in case a Level 0 test failed). Following the standard daily air calibration, the CT technologists were instructed to perform daily QC scans according to the workflow proposed above. They had the responsibility to perform the Level 0 test and review the results, perform the Level 1 test if the Level 0 test failed, and to inform the medical physicist if a Level 1 test was performed. The imaging protocols that were used for the Level 0 and Level 1 tests are presented in Table 2. Initially, the medical physicist reviewed the QC reports from MonitorCT, sent via mail every morning, and contacted the head CT technologist if a Level 0 test failed with the instruction to perform a Level 1 test. After the first year, a Web interface was developed to display the results from the evaluations with MonitorCT whereby the technologists could review the result of the test and perform the Level 1 test if necessary. The Web interface provides current scanner status and support for trend analysis of the KPIs.

**Table 2:** Scan parameters for the Level 0 and Level 1 tests on the connected CT scanners during the pilot study

*Level*	*Scanned Part*	*No. of Slices*	(*kVp)*	*SFOV*	(*mA)*	*Rot time (s)*	*FOV (cm)*	*Im Thk (mm)*	*Coll (mm)*	*Conv. Kernel*
1	Positioning	1	120	Head	260	1	25	5	8×0.625	std
	Water	8	120	Head	260	1	25	5	64×0.625	std
1	Positioning	1	120	Head	260	1	25	5	8×0.625	std
	Water	1	80	Head	260	1	25	5	8×0.625	std
		1	100	Head	260	1	25	5	8×0.625	std
		1	120	Head	100	1	25	5	8×0.625	std
		1	120	Head	180	1	25	5	8×0.625	std
		1	120	Head	260	0.5	25	5	8×0.625	std
		1	120	Head	260	1	25	5	8×0.625	std
		1	120	Head	260	2	25	5	8×0.625	std
		8	120	Head	260	1	25	5	64×0.625	std
		8	120	Head	260	1	25	2.5	32×0.625	std
		4	120	Head	260	1	25	1.25	16×0.625	std
		1	120	Medium	260	1	25	5	8×0.625	std
		1	120	Body	260	1	25	5	8×0.625	std
		1	120	Head	600	1	25	5	8×0.625	std
		1	120	Head	600	1	25	5	8×0.625	edge
		1	140	Head	260	1	25	5	8×0.625	std

## III. RESULTS & DISCUSSION

In the Material & Methods section A of this work, relevant KPIs for CT system performance were identified and their relation to existing fundamental QC parameters used during annual QC was established. A method for incorporating KPIs into periodical routine QC based on the identified KPIs was presented in section B. In section C, the implementation of the methodology for testing on a daily basis in a clinical setting was elaborated. The pilot study was carried out for a period of over two years at the Department of Neuroradiology at the Karolinska University Hospital and included more than 900 daily QC scans from the two CT scanners.

**Figure 3:**
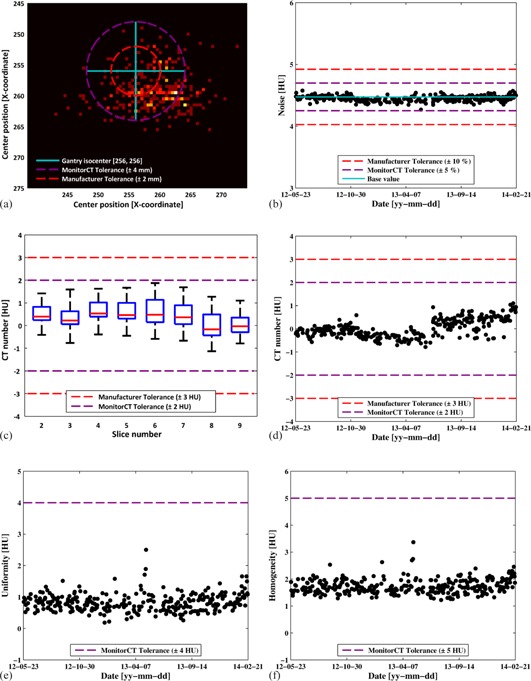
KPIs from one CT scanner's Level 0 tests: (a) distribution of the phantom center (tolerances are for positioning laser accuracy); (b) image noise in one slice; (c) boxplot of CT number of water for all slices (all data points are inside the whiskers); (d) CT number of water in one slice; (e) uniformity in one slice; (f) homogeneity in one slice.

The KPIs from the Level 0 tests for one of the CT scanners are shown in Fig. 3. Figure 3(a), the positioning of the phantom center is presented, revealing a systematic error of 3–4 mm in its position. Following these results, the phantom positioning was investigated. The positioning lasers were tested independently with a Catphan 600 (The Phantom Laboratory, Salem, NY) and found to be within tolerance. It was concluded that the technologists were handling the QA phantom appropriately when positioning with the lasers. However, the deviations in positioning were found to be caused by problems mounting the QA phantom in a precise manner, due to the instability of the phantom bracket. This result shows that the manufacturer's QA phantom offers limited precision in the test of the positioning KPI. It may be of benefit to use another phantom, or design an in‐house phantom, that has high precision in its positioning. Figure 3(b) presents the KPI image noise relative to its base value for one slice position. A boxplot of the KPI CT number of water for all eight slice positions is presented in Fig. 3(c). The boxplot is configured so that all data are inside the whiskers. A trend curve for the KPI CT number of water for one slice location is presented in Fig. 3(d). Figures 3(e) and (f) show trend curves for the KPI uniformity and the KPI homogeneity evaluated in one slice. From the figure it can be seen that the trends in uniformity and homogeneity closely follow each other. A homogeneity test could, therefore, be used instead of a uniformity test, providing additional sensitivity to some forms of artifacts.

**Figure 4:**
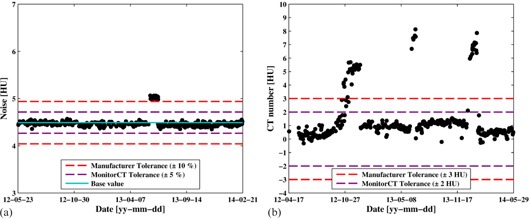
Examples of when the proposed methodology has helped medical physicists in finding deviations in scanner performance: (a) image noise values out of tolerance; (b) CT number of water out of tolerance multiple times.

During the two years of the pilot study, four deviations in scanner performance were found. The deviations are presented in Fig. 4. Figure 4(a) displays the KPI image noise from one slice location in the Level 0 test for one of the CT scanners over a two‐year period. On one occasion, the KPI image noise deviated outside of the allowed tolerance. The deviation was discovered promptly and was caused by a barely visible ring artifact. After discussing the cause of the ring artifact with the manufacturer it was concluded that a small amount of dirt was present in the collimator during the water calibration of the CT system. Ideally, such artifacts should also be spotted with the homogeneity tests, but the initial tolerance level defined for the KPI, 6 HU, was too high and the homogeneity test did not fail. Consequently, the tolerance for the KPI homogeneity was lowered to 5 HU. Test data are being accumulated at our center to establish whether this tolerance is acceptable. The homogeneity test can perhaps never entirely replace human evaluation in the identification of artifacts; however, its potential for rapid and automatic identification of many such aberrations makes it a valuable addition to the available arsenal of QC tests. Figure 4(b) presents a trend curve for the KPI CT number of water for one slice location of the Level 0 test. The KPI CT number of water went outside of the allowed tolerance on three occasions during the pilot study. The manufacturer has verified that the problems were caused by an error related to the air calibration of the system. On all three occasions, the results could not be seen in patient images and the clinic decided to continue scanning patients, with the problem monitored, until the manufacturer came to perform a preventive maintenance. This maintenance included a new water calibration, after which the CT numbers of water returned to their expected values.

The deviations in CT scanner performance that have been presented here were small and had a minor effect on patient images. Without a daily QC routine, most likely, they would not have been discovered until the next annual QC or planned service, or until the radiologists would have noticed degraded image quality in the clinical images. Furthermore, the deviations were discovered promptly due to the high frequency of the KPI tests (sampled daily). This, in turn, entails that the CT system could be serviced appropriately in a timely fashion to prevent identified issues deteriorating into clinical problems. The results from the pilot study demonstrate the utility of testing KPIs on a daily basis. It is therefore recommended to perform daily KPI tests in order to promptly discover and service abnormalities in CT system performance that could potentially affect the clinical outcome of CT patient examinations. The possibility to perform trend analysis of various parameters gave valuable insight into the deviations and helped the medical technicians and the manufacturer to localize and handle the deviations.

The individual KPIs used to discover the four deviations discussed above were the only KPIs to go outside of their allowed tolerances. In the case of the ring artifact which was discovered only by the KPI image noise, it was expected that the KPI homogeneity should go outside of its tolerance, as well. As the sensitivity of the tests using KPIs is restricted by the tolerance levels defined, the user can define how large the deviations that can be accepted from the clinical viewpoint. In other words, tolerances can be tweaked to increase the sensitivity of the individual KPI tests. Continuous data collection of Level 0 and Level 1 tests over larger periods of time will provide data on how sensitive the different KPIs are and what tolerances that are possible, and meaningful, to implement.

**Figure 5:**
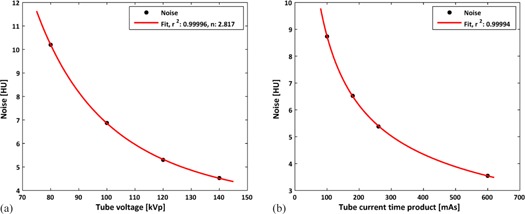
Two examples of analysis that can be performed with a Level 1 test. (a) The KPI image noise versus tube voltage fitted to $\sigma =a\cdot {\left(\text{kVp}\right)}^{n}+c$, where the kilovoltage dependency factor is n=b×2. The slices in this analysis were 2, 3, 7, and 33. (b) The KPI image noise vs. tube current time product fitted to Brooks formula. The slices in this analysis were 4, 5, 7, and 31.

Finally, two examples of analyses a medical physicist could perform using the daily test data to help in detecting and understanding deviations in CT scanner performance are shown in Fig. 5. In Fig. 5(a) the KPI of image noise is plotted at different tube voltages. A fit to the equation $\sigma =a\cdot \;{\left(\text{kVp}\right)}^{n}+c$ was made, resulting in fit‐value of n=2.82 for the kilovoltage dependency factor. In Fig. 5(b), the KPI for image noise is displayed at different tube current time products (mAs). A fit to Brooks formula was made yielding an $r^{2}=0.99$. These data for these analyses was available from a Level 1 test.

As the KPI‐based QC methodology is both fast and simple to use, it has now become an integral and welcomed part of the CT system's daily start‐up routine at the clinic.

## IV. CONCLUSIONS

A set of KPIs for CT system performance that are measurable using a standard manufacturer CT QA phantom were introduced. A methodology to perform routine QC using such KPIs was also presented. The simplicity of the method makes it feasible to perform routine QC on a daily basis. The results are stored in a database for ease of further analyses. The implementation in a clinical setting has been evaluated over a two‐year period, with promising results. The method has on several occasions identified abnormalities in CT performance that would otherwise not have been detected until a later time.

Medical physicists and other professionals concerned with the performance of CT systems will, using the presented daily QC routine, have access to comprehensive data on the current status and long‐term trends of CT systems. Swift and pinpointed actions can then be taken in order to ensure the quality of the CT examinations and patient safety. The ease and short time required to perform this daily routine QC mean that it can readily be implemented in clinical practice once suitable software tools have been developed.

## ACKNOWLEDGEMENTS

This research is part of the XQuality project at Karolinska University Hospital in Stockholm, Sweden.

## Supporting information

Supplementary Material
