# Supplementary figures and images for: Quality control of CT systems by automated monitoring of key performance indicators: a two‐year study
Source: J Appl Clin Med Phys. 2015 Jul 8;16(4):254–65. doi: 10.1120/jacmp.v16i4.5469 (PMC5690007; doi:10.1120/jacmp.v16i4.5469)

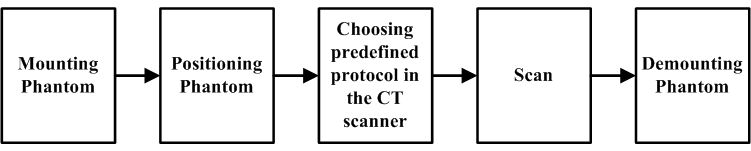

Supplement: Supplementary file 1 — Supplementary Material [file ACM2-16-254-s001.png]
